# Esophageal Adenocarcinoma Presenting as an Isolated Brain Lesion 6 Years After Treatment

**DOI:** 10.14309/crj.0000000000001263

**Published:** 2024-02-17

**Authors:** Jeevan Murthy, John Moise, Kaitlyn Mi, Sonikpreet Aulakh

**Affiliations:** 1 West Virginia University School of Medicine, Morgantown, WV; 2 Vanderbilt University School of Medicine, Nashville, TN; 3 Division of Hematology-Oncology, West Virginia University Cancer Institute, Morgantown, WV

**Keywords:** esophageal adenocarcinoma, brain metastasis, next-generation sequencing, gamma-knife radiation, immunotherapy, neurooncology, gastroenterology

## Abstract

Intracranial metastasis is a rare sequela of esophageal adenocarcinoma (EAC), typically presenting within the first 2 years after primary tumor detection. Our patient is a 72-year-old man diagnosed with an EAC in 2015 and presented with recurrence of a distant solitary brain lesion approximately 6 years after the initial diagnosis. Histological diagnosis was confirmed as EAC with all relevant indicators. In addition, we used genomic profiling to detect biomarkers that can be useful in the future for therapies.

## INTRODUCTION

Esophageal adenocarcinoma (EAC) is among the most common cancer diagnoses in the United States.^1^ Even so, it is becoming ever more prevalent because of risk factors including gastroesophageal reflux disorder, Barrett's esophagus, and obesity.^2^ Common presenting symptoms include progressive solid-to-liquid dysphagia, retrosternal pain, and cachexia. If not detected early, EAC tends to progress and metastasize to the liver, lungs, and regional lymph nodes as most common sites.^3^ Central nervous system (CNS) metastasis is a rare phenomenon among patients with EAC with prevalence of <5%, typically presenting just months after the primary tumor discovery.^4–7^

## CASE REPORT

Our patient is a 72-year-old White man with a medical history including diverticulosis, Zenker diverticulum, and a 40-pack-year smoking history. Family history is unremarkable and does not include gastrointestinal premalignancy, cancer, or other inflammatory disorders.

In March of 2015, the patient presented to a local clinic with several months of progressive dysphagia and unintentional weight loss of forty pounds. Contrast-enhanced computerized tomography of the chest, abdomen, and pelvis revealed a constricting mass at the esophagogastric junction with irregular wall thickening, luminal narrowing, and adenopathy of the gastrohepatic ligament. Endoscopic biopsy of the mass confirmed the diagnosis of EAC, an aggressive form of carcinoma with glandular differentiation. Soon after discovery, the patient began neoadjuvant chemoradiation (consisting of cisplatin and 5-fluorouracil, 45-Gy radiotherapy) with a minimally invasive gastroesophagectomy and a feeding jejunostomy performed in July of 2015. Pathologically enlarged superior abdominal nodes were noted within the resected specimen. The postoperative course was without complication. The final tissue diagnosis was stage IIIBT3N2cM0 EAC.

**Figure 1.**
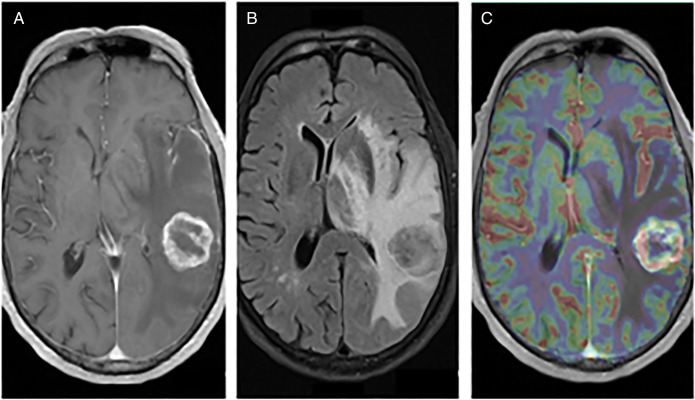
Preop imaging of L parietal lobe mass. (A) T1 axial 1 GD, (B) T2 FLAIR, and (C) T1 OLEA. FLAIR, fluid-attenuated inversion recovery.

**Figure 2.**
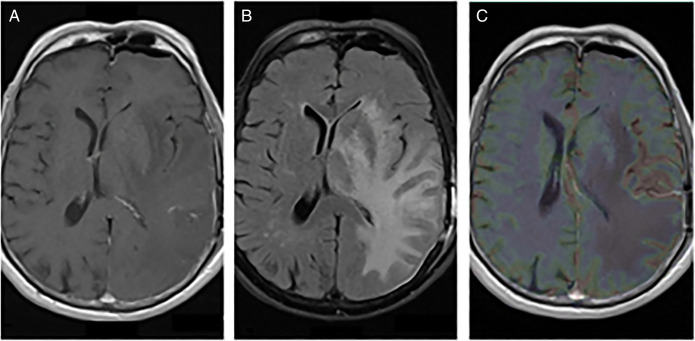
Postop imaging of L parietal lobe mass. (A) T1 axial 1 GD, (B) T2 FLAIR, and (C) T1 OLEA. FLAIR, fluid-attenuated inversion recovery.

**Figure 3.**
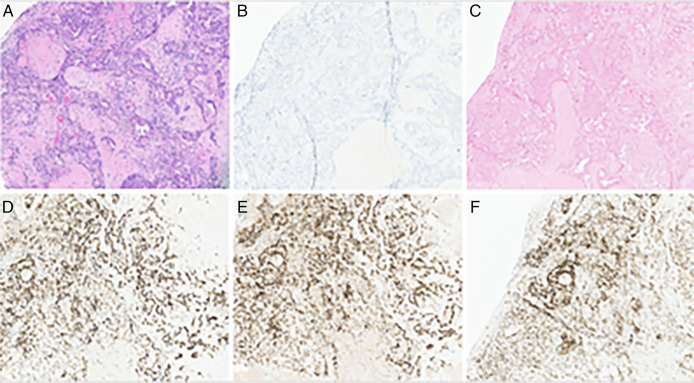
Histological staining of brain metastasis. (A) Hemolysin and eosin staining, (B) HER2-negative, (C) EBER ISH–negative, (D) MSH6-positive, (E) MSH2-positive, and (F) MLH1-positive. EBER, Epstein-Barr encoding region; ISH, in situ hybridization.

**Figure 4.**
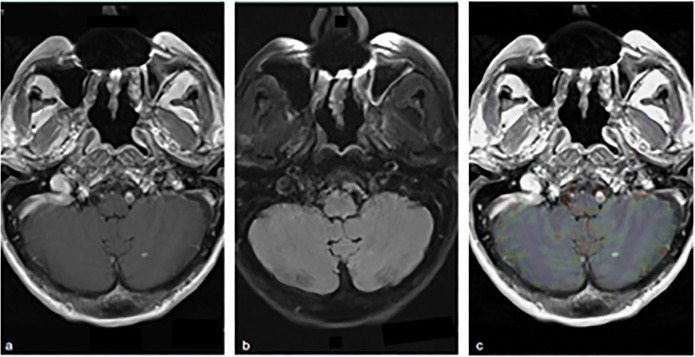
Disease recurrence in L cerebellar hemisphere. (A) T1 axial 1 GD, (B) T2 FLAIR, and (C) T1 OLEA. FLAIR, fluid-attenuated inversion recovery.

Clinical and radiological surveillance over the next 6 years did not show any evidence of disease recurrence. In June of 2021, the patient presented to a local emergency department complaining of 2 months of worsening confusion, dysarthria, and word-finding difficulties. On examination, the patient was awake, alert, and oriented to person and place, although he was poorly oriented to time. A contrast-enhanced computerized tomography of the head revealed a parietal lobe lesion measuring 2.8 × 2.6 × 2.8 cm with involvement of the thalamus and basal ganglia. Also observed was vasogenic edema and an 8.8-mm midline shift. Contrast and perfusion-enhanced magnetic resonance imaging of the brain highlighted a T2-hyperintense left parietal lobe mass (Figure 1). Head-to-toe fludeoxyglucose positron emission tomography was negative for extracranial disease. The tumor was resected through left posterior temporal craniotomy, and the procedure was without complication (Figure 2). Surgical pathology categorized the resected tissue as undifferentiated high-grade epithelial cells with necrosis and glandular proliferation, positive for *MSH6*, *MSH2*, and *MLH1* and negative for *HER2* and Epstein-Barr encoding region in situ hybridization (Figure 3). Thereafter, the patient received 18-Gy gamma-knife radiation to the tumor fossa. Surveillance scans after 1 year including PET and contrast-enhanced magnetic resonance imaging revealed a 4-cm ring-enhanced focus in the left cerebellar hemisphere, which led to additional 22-Gy gamma-knife radiation (Figure 4). Currently, the patient is in complete remission.

## DISCUSSION

Diagnostic pathology confirmed that the resected parietal mass is consistent with the EAC. Tumor sequencing showed a low mutational burden of 4 mutations/Mb, no genomic loss of heterozygosity, and stable microsatellite repeats. Whole-exome sequencing demonstrated well-characterized pathogenic mutations of the *TP53*, *ARID1A*, and *FH* genes, resulting in genomic instability, dysregulation of histone acetylation, and aberrant gene transcription, as well as upregulation of hypoxia-inducible factor–mediated angiogenic transcription pathways, respectively.^8–10^ Furthermore, sequencing identified a previously unreported variant of *CDH1*, a tumor suppressor gene that encodes epithelial cadherin whose loss has been associated with tumor proliferation, invasion, and metastasis.^11,12^ Although the genomic screening detected several possible key driver mutations, the current information cannot be fully relied on to explain the brain prediction as well as latency of the presentation.

Our online database search identified 16 cases of EAC with brain metastasis occurring from 1997 to 2012 (Table 1). Noted sites of intracranial metastasis included the cerebellum, leptomeninges, pineal gland, and limbic system, in addition to various locations throughout the cerebral cortex. Of note, the mean age of brain metastasis presentation was approximately 58.5 years. The latency period from EAC diagnosis to presentation of brain metastasis proved to be variable in the literature, with a mean of 1.9 years and an SD of 2.1 years. None of the cases reported any genetic data of either the primary or secondary tumors.

**Table 1.** Online database search for cases of esophageal adenocarcinoma with intracranial metastasis

PMID	Sex, age at EAC diagnosis	EAC staging	Time to discovery of intracranial metastasis	Locations of intracranial metastasis	Positive genetic markers	Treatment regimen
23606394	M, 74	T3N1M0	3.5 years	R temporal lobe	HER2	Esophagectomy, RT, and met resection
29181738	M, 53	T1bN1M1 stage IV	Concurrent	L parietal lobe	NR	Esophagectomy, RT, and met resection
25436131	F, 55	Stage IVa	1 year	R cerebellum and R parietal lobe	HER2	RT, WBR, and met resection
33490960	M, 53	Siewert II, stage G2T3N2M0 (IIIB)	5 years	Cerebellum	NR	Esophagectomy, GKR, and met resection
31977493	M, 62	NR	6 months	Cerebellum	HER2 (−)	CR
28215583	M, 64	NR	1 year	Pineal region	NR	Esophagectomy, RT, met resection, and CR
26183738	M, 64	NR	4 years	L occipital lobe	NR	Esophagectomy, met resection, and CR
26183738	M, 71	NR	6 years	R temporal lobe	NR	Esophagectomy, met resection, and CR
16434296	M, 53	Stage IVa	6 months	Posterior frontal lobe	NR	Esophagectomy, met resection, and CR
29102948	W, 74	Siewert type 2 tumor, type 3, Tub1-2, pT3 (SS), pN1 and stage IIB	5 months	Cerebellum	NR	Gastrectomy, CR, met resection, and GKR
30956201	M, 28	TXN1M1	Concurrent	Multiple; largest in L parietal lobe	HER2 2+ equivocal	WBR
9105327	W, 51	NR	6 months	Caudate nucleus	NR	Met resection and CR
25503172	M; M, NR	NR	NR	Multiple	HER2	WBR and GKR
31632701	M, NR	NR	NR	IDEM lesion	NR	Met resection
22785960	M, NR	NR	NR	NR	NR	CR

CR, chemoradiation; GKR, gamma-knife radiation; IDEM, intradural extramedullary; NR, not reported; RT, radiation therapy; WBR, whole-brain radiation.

The unusual presentation of this EAC case progressing as a solitary CNS metastasis years later warrants discussion about predictive biomarkers. Precision medicine allows us to use specific genomic targets to guide the therapy, limit the disease burden, and improve patient's overall prognosis. Because there is no current standard-of-care therapy for patients with CNS metastases from EAC, we should consider profiling tumor specimens at early stages of primary disease presentation. In our case, genomic profiling of the metastatic tumor provided an insight into tumor's unique set of gene expression. Our case adds very specific information to the pool of literature in latent CNS metastates from EAC.

Despite an early stage EAC in complete remission, the probability of solitary brain metastasis without the systemic involvement after many years is still a real possibility. Limited data suggest that the survival of patients with isolated CNS lesions is <1 year.^13,14^ Neoadjuvant chemoradiation therapy has shown long-term survival benefits in several studies.^15,16^ However, the current literature lacks information on EAC specific genes that can predict metastases to the brain. We want to highlight the significance of genetic sequencing in early stages of EAC to not only guide adjuvant therapy in the future but to also optimize the duration of the clinical and radiographic surveillance.

## DISCLOSURES

Author contributions: The patient was managed clinically by S. Aulakh. The case and literature review plan were designed and performed by S. Aulakh, J. Murthy, and J. Moise. The manuscript was written and edited by J. Murthy, J. Moise, S. Aulakh, and K. Mi. S. Aulakh is the article guarantor.

Acknowledgement: The authors thank CARIS for providing the genomic and histological data.

Financial disclosure: None to report.

Previous presentation: Presented at the American College of Gastroenterology Annual Scientific Meeting; October 25, 2022; Charlotte, North Carolina.

Informed consent was obtained for this case report.
